# Investigation of the Possible Role of RAD9 in Post-Diapaused Embryonic Development of the Brine Shrimp *Artemia sinica*

**DOI:** 10.3390/genes10100768

**Published:** 2019-09-30

**Authors:** Huifang Huang, Ce Chen, Feng Yao, Xiuling Li, Yanan Wang, Yuting Shao, Xinyao Wang, Xingzheng Zhang, Tao Jiang, Lin Hou

**Affiliations:** 1 School of life sciences, Liaoning Normal University, Dalian 116081, China; 100932@lnnu.edu.cn (H.H.); 18742501207@163.com (C.C.); luckyfrog@126.com (F.Y.); a1055914942@163.com (X.L.); wyn131417@163.com (Y.W.); 13841831939@163.com (Y.S.); wxyao0701@163.com (X.W.); 13478577682@163.com (X.Z.); 2 Department of Biology, Dalian Medical University, Dalian 116081, China

**Keywords:** *Artemia sinica*, *As*–rad9, diapause embryo, cell cycle checkpoint protein, RAD9

## Abstract

Background: The cell cycle checkpoint protein RAD9 is a vital cell cycle regulator in eukaryotic cells. RAD9 is involved in diverse cellular functions by oligomer or monomer. However, the specific mechanism of its activity remains unknown in crustaceans, especially in embryonic diapause resumption of the brine shrimp *Artemia sinica*. Methods and Results: In the present article, a 1238 bp full-length cDNA of *As*–RAD9 gene, encoding 376 amino acids, was obtained from *A. sinica*. The expression pattern of *As*–RAD9 was analyzed by qPCR and Western blot. The mRNA expression level climbs to the top at the 10 h stage of embryo development, while the protein expression pattern is generally consistent with qPCR results. Moreover, the *As*–RADd9 related signaling proteins, *As*–RAD1, *As*–HUS1, *As*–RAD17, and *As*–CHK1, were also detected. Immunofluorescence assay showed that the location of *As*–RAD9 did not show tissue or organ specificity, and the intracellular expression was concentrated in the cytoplasm more than in the nucleus. We also explored the amount of *As*–RAD9 under the stresses of cold and high salinity, and the results indicate that *As*–RAD9 is a stress-related factor, though the mechanisms may be different in response to different stresses. Knocking down of the *As*–RAD9 gene led to embryonic development delay in *A. sinica*. Conclusions: All these results reveal that *As*–RAD9 is necessary for post-diapaused embryonic development in *A. sinica*.

## 1. Introduction

The brine shrimp *Artemia sinica* (arthropods, crustaceans, Branchiopoda, Anostaca, *Artemia*,) is extensively distributed in salt lakes in China. *A. sinica* is an excellent live food for shrimps, crabs, and fishes; hence, it is widely used in aquaculture and experimental research [1]. The diapause of *A. sinica* is one of the defense mechanisms against extreme environments, such as extreme temperature, water shortage, hypoxia, and high salinity. Some studies have shown that *A. sinica* will diapause in low temperature and high salt environment [2,3,4]. At the same time, the metabolic level of embryonic cells is significantly reduced, the cell cycle is arrested, and the life activity is almost completely stopped [5]. This state can be maintained for a long time, so that the *Artemia* cysts can withstand the damage of the harsh environment. After diapause termination, the embryo cells remain in a resting situation and resume developing only if the survival conditions are feasible [2,3,4]. However, the molecular mechanism involved in cell cycle recovery during the restarting of diapause embryo is still unknown, which has become a research hotspot in this field.

Eukaryotic cell division can be delayed at cell cycle checkpoints because of cellular damage, exogenous stress signal, nutrients, or essential growth factors [6,7]. Proteins that function in the regulation of cell cycle checkpoint are critical for fate determination in different cells. RAD9 was first discovered in cell–cycle arrest induced by DNA damage in budding yeasts [8,9]. Deletion of RAD9 in mice showed checkpoint abnormalities and embryonic lethality [10]. Recent studies found that RAD9is a versatile gene which is associated with apoptosis, DNA repair, genome integrity, cell cycle checkpoint control, radioresistance, transactivation of downstream genes, ribonucleotide metabolism, and telomere maintenance [8,11,12]. RAD9 interacts with a multitude of proteins directly or indirectly, which are involved in diverse physiologic functions. The indirect interaction is mostly mediated by the RAD9, RAD1, and HUS1 (9–1–1) complex. Extracellular stress-induced DNA damage can be repaired through the ataxia telangiectasia mutated (ATM) and ATR (ATM and RAD53-related) signaling pathways. At the same time, the 9–1–1 complex is phosphorylated by the loader complex RAD17–replication factor–C, and transferred to the impaired position in a replication protein A (RPA) dependent method, and then the heterotrimeric slides along DNA as a processivity factor for DNA polymerases [11]. The 9–1–1 complex can also bind to RHINO (RAD9, RAD1, Hus1 interacting nuclear orphan) [13,14] and TopBP1 [15] to further amplify the ATR signal, and participate in the activation of different downstream substrates, including CHK1, thereafter activating downstream effectors such as CDC25A and WEE1 [16,17]. In mammals, phosphorylation of RAD9 mediates the binding of 9–1–1 to TopBP1 and activates the ATR–CHK1 checkpoint pathway [18].

However, little research has been done on arthropods; in particular, the molecular mechanism and function of RAD9 are still unknown in *A. sinica*. In this study, the full-length cDNA of *As*–RAD9 from *A. sinica* was cloned. Real-time qPCR and Western blotting were used to analyze the mRNA and protein expression levels during embryonic development. The expression location of *As*–RAD9 was determined by immunofluorescence assay. The morphological changes and mRNA expression after interference were observed by siRNA interference assay. Our aim is to further explore the role of *As*–RAD9 in the early embryonic development of *A. sinica* and response to exogenous stresses like cold and high salinity.

## 2. Results

### 2.1. Cloning and Bioinformatic Analysis of As–RAD9

The 1238 bp full-length cDNA of *As*–RAD9 (GenBank accession number: MH797557) was obtained, which contains an 1131 bp open reading frame (ORF), and the length of 5′–UTR and 3′–UTR were 3 bp and 107 bp, respectively (Figure S1a). The encoding protein contains 376 amino acids, whose predicted molecular weight is 42.6 kDa, and the pI is 6.48. *As*–RAD9 has 38 phosphorylation sites, indicating that it may frequently suffer post-translational modification (Table 1). The subcellular prediction showed that *As*–RAD9 was most likely (60.9%) to be in the nucleus, 30.4% in the mitochondria, and 8.7% in the cytoplasm. The *As*–RAD9 is a non-secretory protein, since the SignalP 4.0 fails to find a signal peptide in the protein. Protscale indicated that the putative protein was most likely to be hydrophilic (MIN: −3.100, MAX: 2.011). The TMHMM Server 2.0 (http://www.cbs.dtu.dk/services/TMHMM/) showed that *As*–RAD9 has no transmembrane helices, indicating that *As*–RAD9 is not a transmembrane protein. 

The homology analysis of the *As*–RAD9 protein sequence revealed a highly conserved amino acid sequence between *A. sinica* and the other 12 species of GenBank (Figure S2); the result of multiple sequence alignment was further used for phylogenetic tree construction and evolutionary relationships analysis. The neighbor-joining (NJ) tree was built in Mega 4.1 software [19], with a bootstrapping value of 1000 (Figure S3).

### 2.2. Expression of As–RAD9 by qPCR

**Figure 1:**
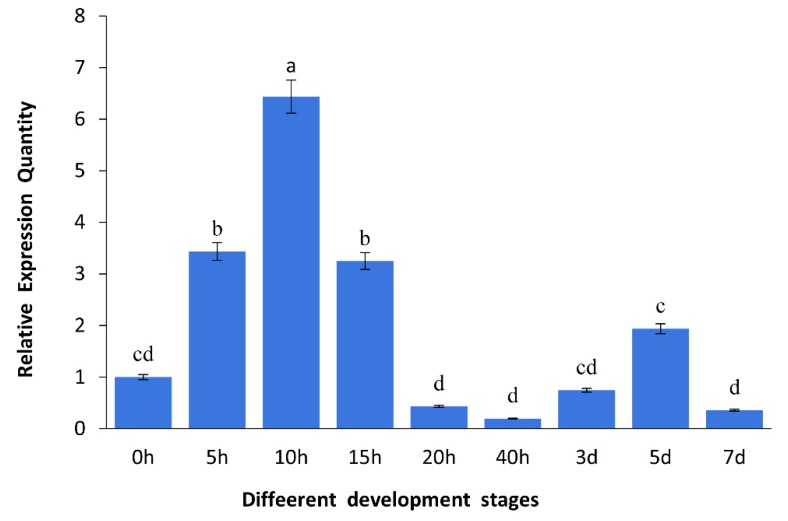
Quantitative real-time qPCR analysis of the expression levels of *As*–RAD9 in different developmental stages of *A. sinica*. The 0h stage expression level of *As*–RAD9 was set as a control group, and the mRNA expression levels of *As*–RAD9 at different time points during development was measured. The x-axis represents the various phases (0 h to 7 days), and the y-axis represents the expression level relative to 0 h. Data are mean ± SD of three replicate experiments, and significant differences in different developmental stages (*P* < 0.05) were analyzed by one-way ANOVA and are indicated by lower case letters (a–d).

**Figure 2:**
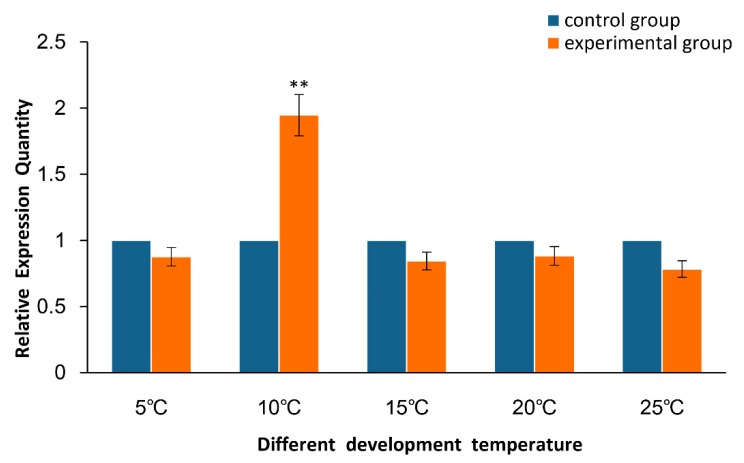
Quantitative real-time qPCR analysis of the expression level of *As*–RAD9 in different challenge temperatures. The expression level of *As*–RAD9 mRNA at 30 °C was set as a control group, and the expression level of *As*–RAD9 mRNA at five different temperatures was measured. Data are mean ± SD of three replicate experiments, and ** indicates a very significant difference compared to the control (*P* < 0.01).

**Figure 3:**
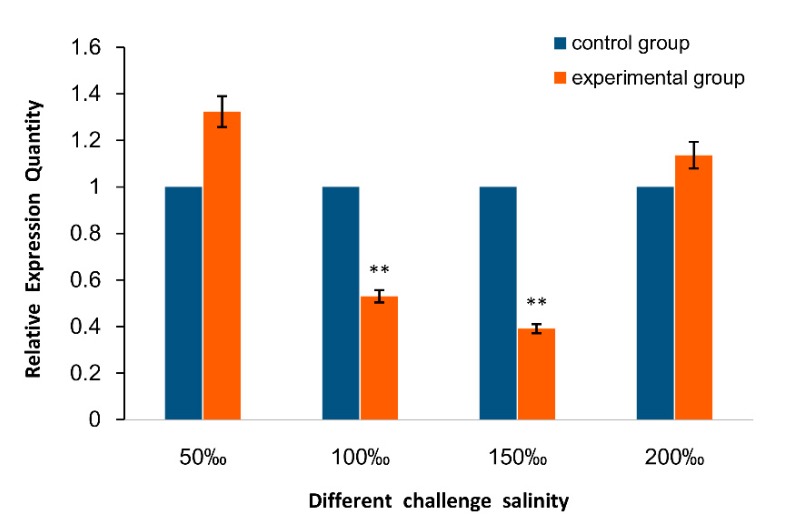
Quantitative real-time qPCR analysis of the expression levels of *As*–RAD9 in different salinity challenge. The expression level of *As*–RAD9 mRNA was adjusted to the control group at 28‰ salinity, and the expression level of *As*–RAD9 mRNA was measured at four different salt concentrations. Data are mean ± SD of three replicate experiments, ** indicates a very significant difference compared to the control (*P* < 0.01).

The mRNA expression patterns of *As*–RAD9 were tested by qPCR in different developmental stages of *A. sinica* (Figure 1). Results displayed that the amount of *As*–RAD9 mRNA began to increase rapidly from 0 h to 5 h at the developmental stage and reached a significantly high level at 10 h. Then, it declined from 15 h and maintained a relatively lower expression level. The expression levels of *As*–RAD9 in the different challenge of temperature (Figure 2) and salinity (Figure 3) in *A. sinica* were also detected. The expression levels of *As*–RAD9 mRNA peaked at 10 °C, while there was no significant difference between other challenges and control group. On the other hand, the expression tendency of *As*–RAD9 under stress of salinity is quite different. The expression level of *As*–RAD9 increased slightly at the tender stress of salinity (50‰), and then slowly decreased with the increase of salinity stress. When the salinity reached 150‰, the amount of *As*–RAD9 mRNA was the lowest, while the amount of *As*–RAD9 mRNA rebounded at 200‰.

### 2.3. Purification and Expression of As–RAD9 Protein

**Figure 4:**
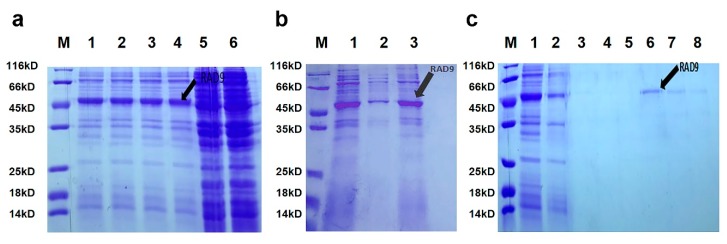
Expression and purification of the *As*–RAD9 fusion protein. (**a**) Different induction treatments of the *As*–RAD9 fusion protein. M: protein markers (14–116 kDa). Lanes 1–4: 1 mM IPTG at 37 °C and 30 °C; 0.25 mM IPTG at 37 °C and 30 °C, respectively, Lane 5–6 (control): non-induced pET–28a–RAD9 cells; Induced pET–28a cells. (**b**) Ultrasonication results of the recombinant pET–28a–Rad9 cells. Lane 1: Total proteins; Lane 2–3: Supernatant and sediment after ultrasonication and centrifugation of induced pET–28a–Rad9 cells; (**c**) Purification of the recombinant *As*–RAD9 protein. Lane 1: Total proteins extracted from induced pET–28a–RAD9 cells; Lane 2: flowed through proteins; Lanes 3–8: elution with 10 mM, 20 mM, 40 mM, 60 mM, 80 mM, and 100 mM imidazole.

The *As*–RAD9 mRNA was cut by EcoRI and SalI, then ligated overnight. The recombinant plasmid pET28a–RAD9 was successfully constructed and confirmed by sequencing. The large-scale purification was conducted at the optimal condition of 1mM isopropyl β- D-thiogalactoside (IPTG) at 37 °C (Figure 4). The *As*–RAD9 recombinant protein was first dissolved in 60 mM urea, then purified using ProteinIso^®^ Ni-NTA Resin column (TransGen, Beijing, China), and finally harvested after dialysis. The molecular weight of the fusion protein was approximately 47 kDa, agreeing with the ProtParam result.

### 2.4. Expression Pattern of As–RAD9 Protein

**Figure 5:**
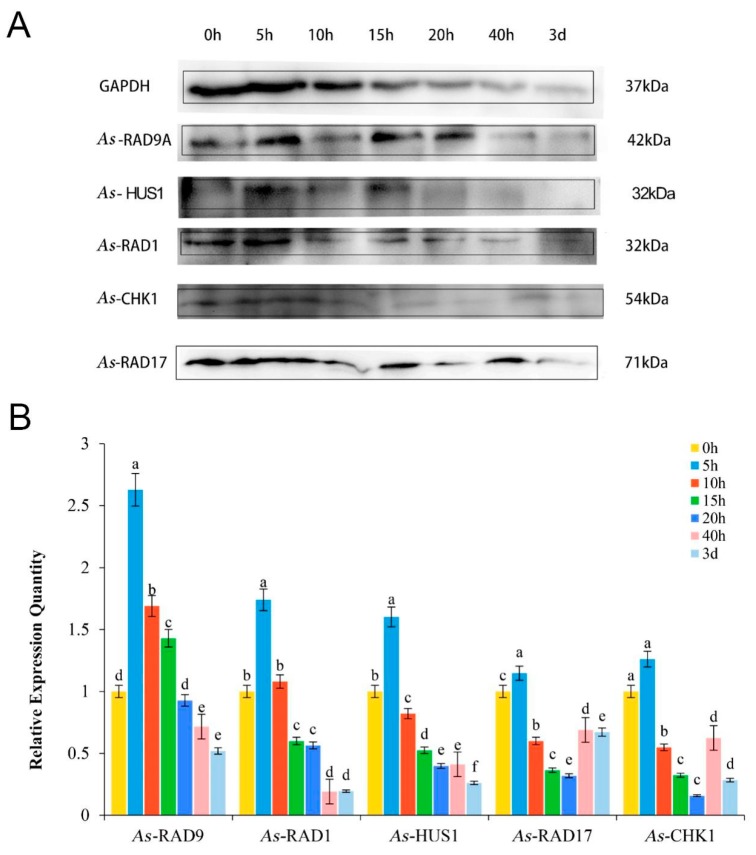
Western blot analyses of *As*–RAD9, *As*–RAD1, *As*–HUS1, *As*–RAD17, and *As*–CHK1 at different developmental stages (0 h–3 days) of *A. sinica*. (**A**) The intensities of the protein bands were normalized against those of GAPDH. (**B**) Values are expressed as arbitrary units of relative value. The x-axis indicates the different protein; the y-axis shows the relative expression level. Significant differences at different development stages (*P* < 0.05) were analyzed by one-way analysis of variance (ANOVA) and reported by lowercase letters (a–e).

The expression of RAD9 in different embryonic development stages of *A. sinica* was detected by western blotting and compared with other four related proteins (*As*–RAD17, *As*–RAD1, *As*–HUS1, *As*–CHK1) (Figure 5). The intensity was normalized to glyceraldehyde-3-phosphate dehydrogenase (GAPDH). The expression level of *As*–RAD9 increased from 0h to 5h at different developmental stages and reached the highest level. Since then, it gradually decreased in expression after 10 h. The other four cell cycle regulatory proteins associated with *As*–RAD9 showed a similar trend to *As*–RAD9.

**Figure 6:**
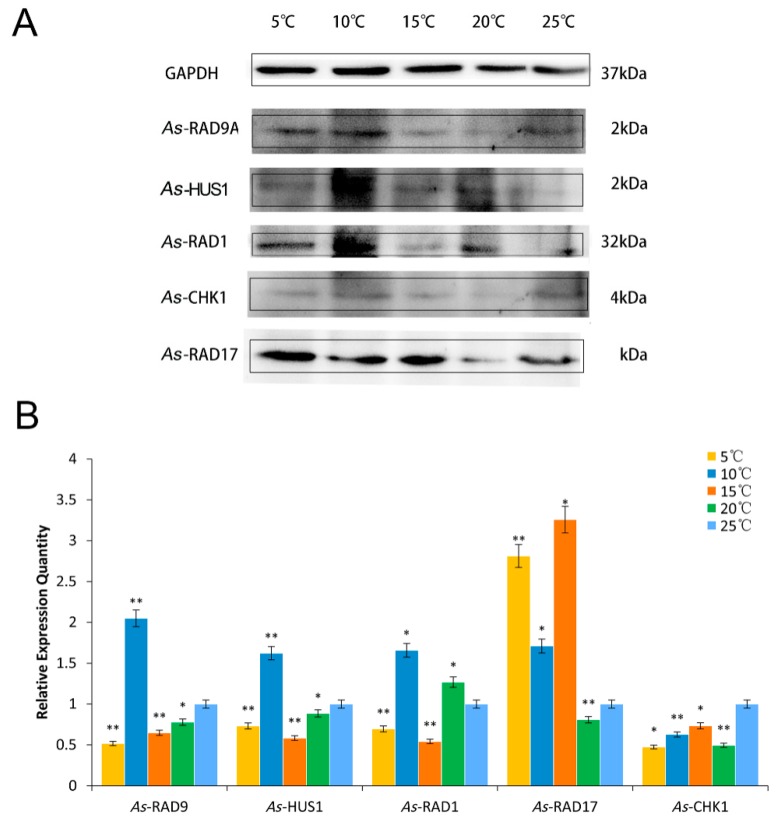
Western blot analyses of *As*–RAD9, *As*–HUS1, *As*–RAD17, *As*–RAD1, and *As*–CHK1 proteins in response to temperature stresses. (**A**) The band intensity of the proteins is normalized to the band intensity of GAPDH. (**B**) The values are expressed as an arbitrary unit of relative value. Protein expression at 25 °C as control (blue), asterisk (**) indicates a statistical difference at *P* < 0.01, and (*) indicates 0.01 < *P* < 0.05.

**Figure 7:**
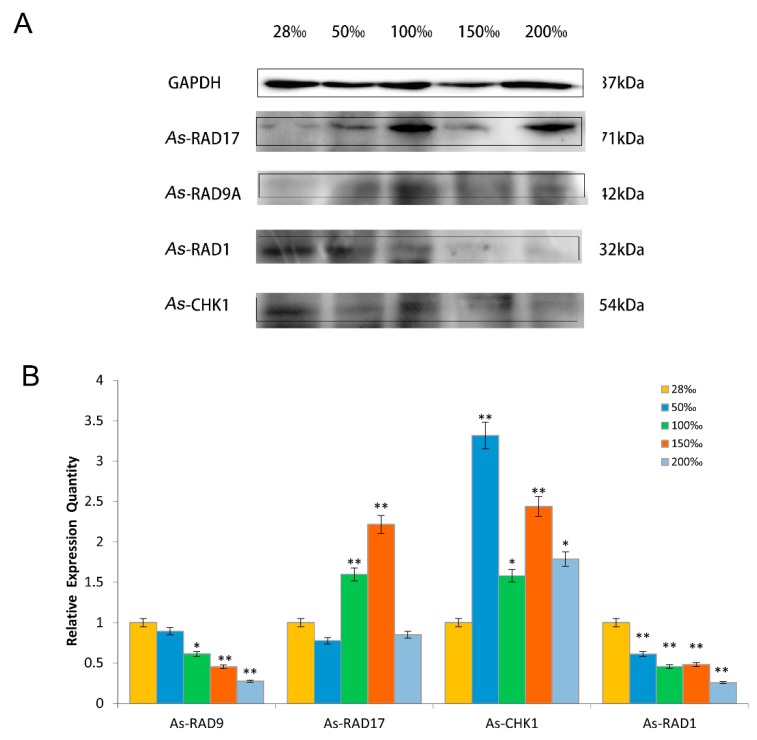
Western blot analyses of *As*–RAD9, *As*–RAD17, *As*–RAD1, and *As*–CHK1 proteins of *A. sinica* under different salt concentration stresses. (**A**) The band intensity of the protein is normalized to GAPDH. (**B**) The values are expressed as an arbitrary unit of relative value. The expression of the protein at a salinity of 28‰ as control (yellow), and asterisk (**) indicates a statistically significant difference of *P* < 0.01, and (*) indicates a 0.01 < *P* < 0.05.

Protein expression patterns of signaling proteins in different challenges of temperature and salinity were performed. In the stress test of different temperatures, the expression levels of *As*–RAD9 protein decreased as the temperatures declined from 25 °C to 15 °C. Then it showed an increasing trend when the temperatures continued descending, with the highest level appeared at 10 °C. Cell cycle regulatory proteins showed a similar pattern to *As*–RAD9. Moreover, with the decrease of temperature, the downstream signaling protein *As*–CHK1 showed a downward trend, and the expression level of the upstream protein *As*–RAD17 showed an upward trend and reached a peak at 15 °C (Figure 6). When it comes to the stress of salinity, the protein expression pattern was quite different. By this time, the expression levels of *As*–RAD9 protein went descending with the increase of salt concentration, and reached the minimum at the salt concentration of 200‰. Besides the 9–1–1 component *As*–RAD1, the expression pattern of cell cycle regulatory proteins (*As*–RAD17 and *As*–CHK1) was opposite to that of RAD9 (Figure 7).

### 2.5. Immunofluorescence Analysis of As–RAD9

**Figure 8:**
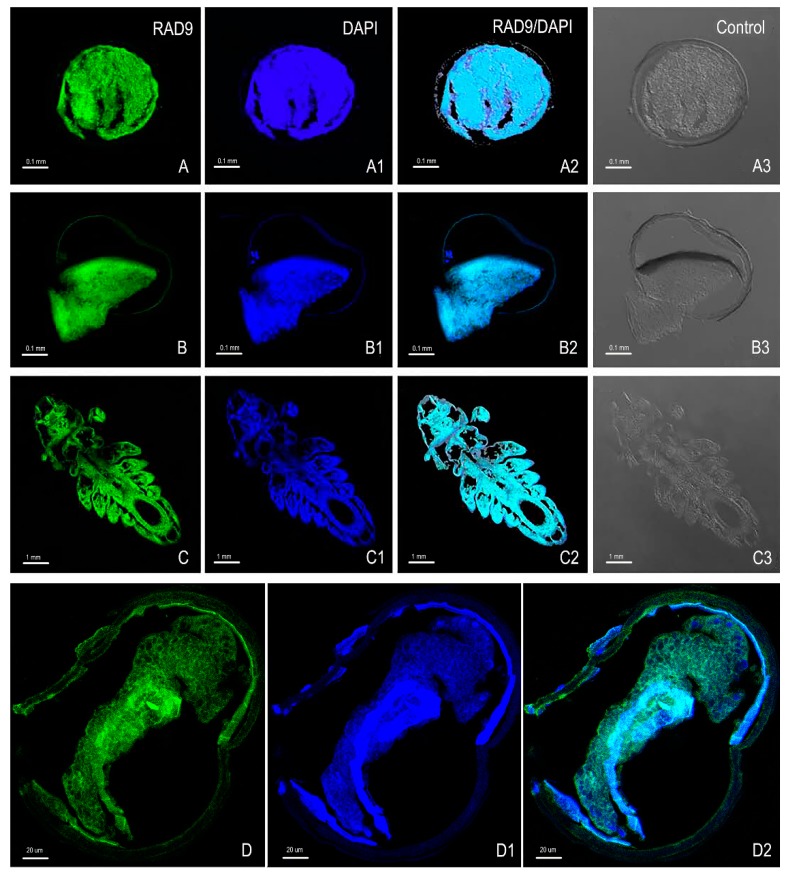
Immunofluorescence analyses of *As*–RAD9 at the embryo and adult stages of *A*. sinica. The paraffin sections of different development stages were prepared for immunofluorescence microscopy. **A** (0 h), **B** (15 h), **C** (7 days), and **D** (10 h) represent single-labelled with polyclonal anti-RAD9; **A1**, **B1**, **C1** and **D1** represent single-labelled DAPI (nuclear blue fluorescent probe); **A2**, **B2**, **C2**, and **D2** represent with the image overlay of control group samples dual-labelled with polyclonal anti-RAD9 and DAPI; **A3**, **B3**, and **C3** represent the image overlay of the control group samples single-labelled with secondary antibody.

To observe the localization of *As*–RAD9, we found embryos and adults using an immunofluorescence (IF) microscopy. The nucleus of *A. sinica* was labelled with 2-(4-Amidinophenyl)-6-indolecarbamidine dihydrochloride (DAPI) in blue fluorescent, and the *As*–RAD9 was detected by fluoresceine isothiocyanate (FITC)-labeled secondary antibody in green. The combined images of the two fluorescences (Figure 8(A2,B2)) were displayed. The results showed that the reactivity of *As*–RAD9 was observed in the whole body of both embryo and adult stages, and the cytoplasmic–nuclear distribution is partly opposite to the prediction results.

### 2.6. Small RNA Interference of As–RAD9

**Figure 9:**
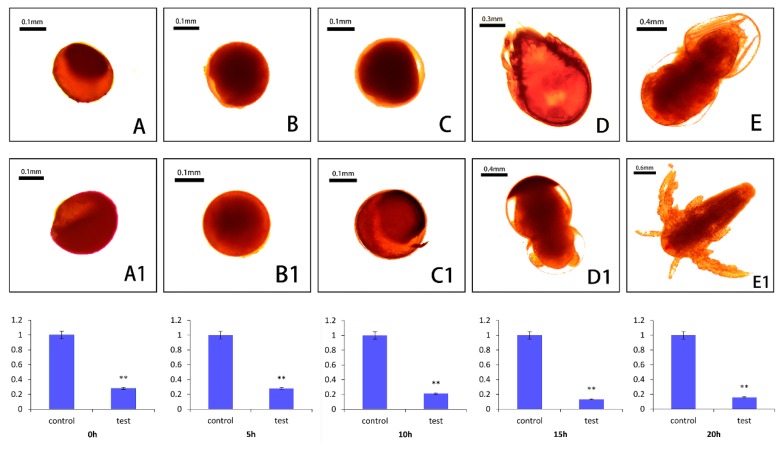
Relative expressions of *As*–RAD9 at different stages after adding the RNAi probe. **A**–**E** represents the experimental group; **A1**–**E1** represents the control group. The qPCR analyses of *As*–RAD9 expression levels were shown below. Data are mean ± SD of three replicate experiments, * indicates a significant difference from the control (*P* < 0.05), and ** indicates a very significant difference compared to the control (*P* < 0.01).

To further explore the role *As*–RAD9 in the post-diapause embryonic development of *A. sinica*, siRNA was used to knock down the expression of *As*–RAD9. Microscopic examination showed that after knocking down of *As*–RAD9, there was no significant difference between the experimental group and the control group from 0h to 5h. The main difference occurred after 10 h, especially when the *A. sinica* developed to nauplii, the individual growth and development rates were significantly slowed down, and both the abnormal rate and mortality increased (Figure 9A). The mortality rate of the control group was about 23.94%, while the mortality rate of the experimental group was up to 41.23%. The expression levels of *As*–RAD9 mRNA was confirmed by qPCR analysis, revealing that expression of RAD9 in the experimental group was less than that of the control group (0, 5, 10, 15, and 20 h; Figure 9B).

## 3. Discussion

**Figure 10:**
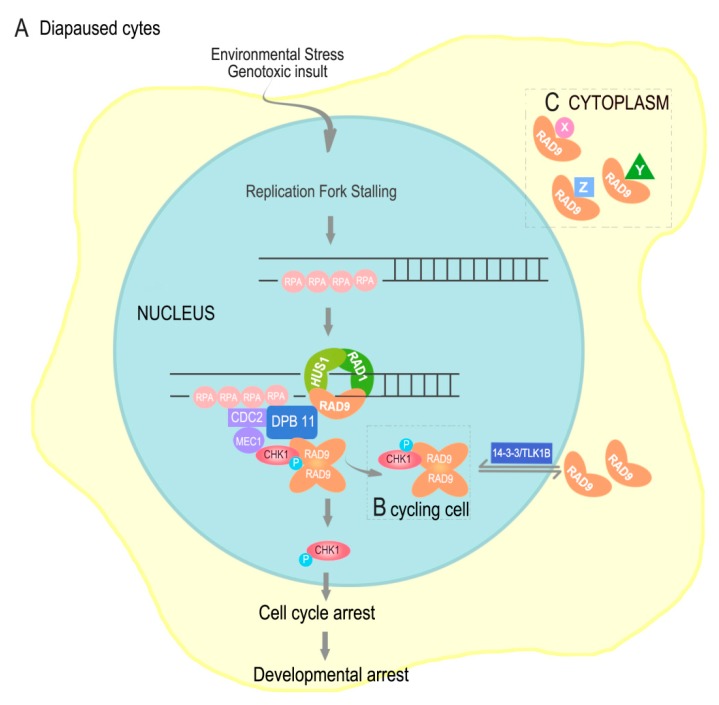
The different functions that *As*–RAD9 may be involved in. (**A**) The checkpoint signaling pathway that may lead to cell and organic developmental arrest; (**B**) The CHK1/RAD9 complex in cycling cells; (**C**) Monomer *As*–RAD9 may interact with different proteins and be involved in various signaling pathways.

The embryonic diapause of *A. sinica* is a programmed developmental arrest [5]. During the diapause embryo restarting, the cell cycle recovery may need a lot of cell cycle checkpoint proteins. RAD9 plays a key role in cell cycle control, mediating cell cycle progression delay or exit in DNA damage or other physiologic stresses. [9,20,21,22]. In the present study, the full-length cDNA sequence of RAD9 from *A. sinica* was cloned for the first time. *As*–RAD*9* is a small gene with a full length of only 1238 bp cDNA, encoding a protein of 376 amino acids. *As*–RAD9 belongs to the RAD superfamily and is an important cell cycle checkpoint protein that contains Bcl–2 homeodomain 3 (BH3) [23,24]. According to the Signal P and Protscale analysis, *As*–RAD9 lacks any signal peptide sequence and transmembrane region, indicating that *As*–RAD9 may not be a secretion protein. Followed immunolocalization analysis showed that *As*–RAD9 is widely distributed in all parts, but sometimes only in the cytoplasm or nucleus, which may be correlated with the different functions of RAD9. The translocation of *As*–RAD9 may rely on nuclear-cytoplasmic shuttling proteins like 14–3–3 and TLK1B [25,26] (Figure 10). The immunolocalization results also showed that *As*–RAD9 was expressed in *A. sinica* at different developmental stages, suggesting that the regulation of *As*–RAD9 may not be restricted by organ specificity.

The development of *A. sinica* includes four main stages: 0–10 h corresponding to the embryo stage, 15–20 h corresponding to the nauplii stage, 40h corresponding to the metanauplius larval stage, and 3 days to 7 days corresponding to the pseudo–adult stage [3]. Real-time qPCR and Western blotting of *A. sinica* embryos at different developmental stages showed that the level of *As*–RAD9 transcript was up-regulated from 0 to 10 h and peaked at 10 h. However, after 10 h, the expression level of *As*–RAD9 gradually decreased and reached the lowest value at 40 h. The results indicate that *As*–RAD9 plays an essential role in the early embryonic stage, which is the most active stage of cell division and differentiation. Our previous studies found that after diapause termination, the embryo cells remain in a resting situation [2,3,4], and the *Artemia* cysts cannot resume development without appropriate conditions. The resumption of resting embryo cells may be involved in a cell cycle control mechanism, which is associated with the extracellular and endogenous cell signaling. RAD9 was found to play a vital role in the G1 cell cycle arrest [9] and the G2/M checkpoint exit [21] in response to environmental stresses, which corresponds to the cell cycle arrest occurred in the transitions of G1/S or G2/M during diapause [5,27]. The rising expression of RAD9 in early embryonic development of *A. sinica* may be associated with the exit of cell cycle checkpoint or maintaining genome stability under the expanding stress of rapid DNA replication during diapause embryo resumption.

To further understand the functions of *As*–RAD9, the 9–1–1 component HUS1, RAD1, and the signaling proteins RAD17 and CHK1 during the early embryonic process of *A. sinica* were analyzed. Western blot results revealed that the expression levels of these proteins increased significantly at 0–5 h, which may be due to the rapid embryo reactivation, accelerated mitosis, expanded DNA replication, and RNA transcript, subsequently leading to an increase in the expression of cell cycle checkpoint control proteins. After 10 h, the mitosis process was stable, and the DNA damage or mispairing may be weakened. Therefore, the expression levels of these proteins were decreased. After 15 h, the *Artemia* larvae resumed growth and development, and the gene expression was further down-regulated. However, at 40 h, the expression levels of RAD17 and CHK1 increased. It is hypothesized that during the rapid development of the pseudo–adult, the synthesis of macromolecules like DNAs, RNAs, and proteins were greatly expanded, the regulation of cell cycle progression must be more complicated, and the role of 9–1–1 complex maybe not so crucial in the normal development as in the embryo restarting of *A. sinica*. 

The evolution strategy for *A. sinica* living through the disadvantageous conditions benefits from the ability of females that produce diapause cysts before the onset of adverse environmental challenges, such as fluctuation of salinity and temperature due to oncoming winter [27,28,29]. To explore the role which RAD9 may be involved in during natural occurrence of diapause, in the present study, the high salinity and low-temperature conditions were repeated to study the expression of *As*–RAD9 in these stress processes. As the temperature decreased to 10 °C, the expression level of *As*–RAD9 was significantly increased, indicating that RAD9 might be involved in the cold response signaling pathway, and 10 °C might be a trigger condition for the corresponding mechanism. On the other hand, with the increase of salinity, the expression of *As*–RAD9 decreased and ultimately rebounded at the salinity of 200‰, indicating that the signaling pathways may be different, since the *Artemia* has to face diverse environmental stresses. When the salinity increased to 200‰, the survival rate of *A. sinica* was meager, and the DNA replication stalling must have been so serious that the checkpoint signaling pathway would be recommissioned to maintain genome stability, so that the expression level of *AS*–RAD9 rebounded. Moreover, the RAD9 is a versatile protein that interacts with a great diversity of proteins by monomer or polymer [11,13] (Figure 10). The different signaling pathways may be associated with complex protein–protein interaction or posttranslational modifications like ubiquitination, phosphorylation, and methylation in response to environmental changes [8,21,22,30,31,32,33], though the specific regulatory mechanism remains to be determined. 

Knockdown of RAD9 in human and mouse cells with different RAD9 states results in extreme cellular sensitivities to various radiations and chemicals [10,34]. Deficient RAD9 and HUS1 in *Leishmania* led to significant opposite phenotypes, which suggests that RAD9 and HUS1 may display different functions in cell cycle control [35]. To verify the effect of *As*–RAD9 deletion on *A. sinica*, we performed siRNA interference experiments. The results showed that knocking down of the *As*–RAD9 gene would reduce the transcript level of *As*–RAD9 and delay the embryonic development process of *A. sinica*. These results suggested that *As*–RAD9 plays an essential role in the early development of *A. sinica*.

In conclusion, the *As*–RAD9 protein may be involved in different signaling pathways during diapause occurred and post-diapause development in *A. sinica*. The stalled DNA replication fork, caused by environmental stress or genotoxic insult, may invoke the checkpoint signaling pathway (Figure 10A), in which RAD9, HUS1, and RAD1 can form a complex (9–1–1) and load onto the damage sites by RAD17. Then, the RAD9 would be phosphorylated and recruited to DPB11, the new 9–1–1–DPB11 complexes activate the MEC1 bound to RPA-coated ssDNA [36], further phosphorylate and remodel both RAD9 and CHK1, which in turn facilitate in cis autophosphorylation of CHK1, subsequently releasing the RAD9/CHK1 complex from the injured ssDNA. Phosphorylation of CHK1 can lead to cell cycle arrest, which may be involved in embryonic diapause, although the mechanism is still obscure. The RAD9/CHK1 complex is constitutive and exits in cycling cells (Figure 10B), also participating in DNA damage recovery and recruited by other RAD9 complex [26]. The immunofluorescence assay indicated that *As*-–RAD9 was distributed more extensively in the cytoplasm than in nucleus, which may be due to the translocation of shuttling proteins like 14–3–3 and TLK1B [25,26]. Besides this, the *As*–RAD9 protein may function as monomer or oligomer in the cytoplasm (Figure 10C). Actually, there are a lot of proteins that interact with RAD9 directly in diverse physiological functions, like P53, TRF2 (telomere integrity), CAD (ribonucleotide synthesis), Bcl–2 (apoptosis), and so forth [12]. In one word, the mechanism of diapause occurred and broken is complicated and may be involved with multiple genes and signaling pathways. Our discoveries are the tip of the iceberg and need further research in the future.

## 4. Materials and Methods 

### 4.1. Preparation of Animal

The collection and preparation of *A. sinica* samples were carried out according to our previous studies [28,37].

### 4.2. Cloning the Full-Length cDNA of As–RAD9

Total RNAs were isolated from the *A. sinica* 0 h cyst by a Trizol kit (Thermo Fisher, Shanghai, China) and reverse transcribed into cDNA according to the supplier’s instructions. Primers were designed based on the *Artemia franciscana* EST sequence in GenBank, and synthesized by Sangon (Shanghai, China) (Table 2). The RT–PCR condition was 94 °C for five minutes, followed 36 cycles of amplification (94 °C for 30 s, 55 °C for 30 s, 72 °C for 45 s), and a final extension at 72 °C for 10 min. The PCR product was isolated, purified, and cloned into the pMD–19T vector and then sequenced by Takara (Dalian, China). Therefore, the 473 bp EST sequence of *As*–RAD*9* was obtained.

The full-length sequence of *As*–RAD*9* was cloned according to the protocol of the SMART RACE cDNA kit (Clontech, Chicago, IL, USA). The gene-specific primers are shown in Table 2. The RACE–PCR product was purified and sequenced by Takara. The sequencing fragments were assembled by DNAMAN 7.1.0 (Lynnon Biosoft, San Ramon, CA, USA); and the resulting full-length cDNA sequence of *As*–RAD9 was submitted to GenBank (accession number: MH_797557).

### 4.3. Bioinformatic and Biostatistics Analyses

The identity and similarity of the nucleotide sequences of *As*–RAD9 were analyzed by BLASTX in the National Center for Biotechnology Information (NCBI) [38]. The ORF was identified using the ORF Finder [39]. The PROSITE tool [40] and SMART [41] were used to predict the structure and functional domains of *As*–RAD9 protein. The molecular weight of the protein and the theoretical isoelectric point (PI) were analyzed by ExPASy’s ProtParam tool [42], and the PSORT and iPSORT service [43] were used to predict the protein subcellular localization. Prediction of transmembrane helices and hydrophilicity were conducted by the Tmpred program [44] and the TMHMM Server 2.0.; Homology analysis of the RAD9 amino acid sequences was performed by the ClustalX 2.0, and MEGA 4.1 was used to further construct the Adjacency (NJ) phylogenetic tree (bootstrapping = 1000). Significant test was analyzed by *t* test using SPSS 18.0 software, and the significance was set to *P* < 0.05.

### 4.4. Expression Pattern of As–RAD9 by qPCR

#### 4.4.1. Expression of As–RAD9 in Early Embryo Development

The cDNA templates were prepared at the same concentration using the method mentioned above. The qPCR primers of *As*–RAD9 and β–actin (inner reference) are listed in Table 2. The qPCR was performed in triplicate for each sample using TB Green Premix Ex Taq (Takara, Dalian, China) and Takara detection system TaKaRa TP800 (Takara, Dalian, China). The reaction procedures were initial denaturation at 95 °C for 30 s, then 38 cycles (95 °C for five seconds, 58 °C for 30 s, 95 °C for 15 s, 60 °C for 30 s) [28]. Based on the Ct values of *As*–RAD*9* and β–actin, the data were analyzed using the comparative cycle threshold (Ct) method (2–Ct^ΔΔ^ method) to calculate the fold increase. Data obtained from real-time qPCR analysis were analyzed by least-squares difference (LSD).

#### 4.4.2. Temperature and Salinity Stress Assays

The cDNA templates were prepared from *A. sinica* (20 h) in the Nauplius stage, which was incubated at the different gradients of temperatures and salinity as described in our previous studies [4,28], and qPCR was performed to examine relative expression values.

### 4.5. Purification of Recombinant As–RAD9 Protein

The complete ORF of *As*–RAD9 was obtained using primers containing specific enzyme sites of EcoRI and SalI (Table 2). The purified PCR product and pET–28a vectors were cut by the two enzymes of EcoRI and SalI, and ligated overnight at 16 °C by T4 DNA ligase (Takara, Dalian, China). After sequencing, the expression vector pET–28a–RAD9 was confirmed inerrably and transferred into competent cell BL21 (DE3) for further induction. Expression of the *As*–RAD9 fusion protein was induced for three hours by four different conditions: 0.25 mM or 1 mM IPTG at 37 °C; 0.25 mM or 1 mM IPTG at 30 °C. Cells were harvested and washed three times with PBS, then collected by centrifugation and sonication. Purification of the *As*–RAD9 fusion protein was performed by Ni–NTA Resin column (Transgen, Beijng, China) according to the supplier’s protocol. Different imidazole concentrations were tested, and 60 mM was selected as the optimal concentration for purification. Proteins were then dialyzed in 20 mM Tris–HCl and fractions were collected and detected by SDS–PAGE and his–Tag western blotting.

### 4.6. Production of Polyclonal Antibodies

Polyclonal antibodies against *As*–RAD9 fusion protein were prepared in rabbits, as described in our previous study [45]. The specificity of the antibody for the purified protein was verified using western blotting.

### 4.7. Western Blotting

Animal samples were prepared from different developmental stages, different temperatures, and salt concentrations. Total proteins were extracted from each sample using RIPA lysis buffer and quantified by the BCA protein assay kit. Protein samples (80 μg each) were subjected to fractionation by SDS–PAGE and transferred to Polyvinylidenefluoride (PVDF) membranes. The PVDF membrane was blocked with 5% skim milk powder for two hours at room temperature and allowed to incubate with the original antibody overnight at 4 °C. Rabbit anti–*As*–RAD9 polyclonal antibody and GAPDH antibody were diluted 1:500 and 1:1000 with PBST, respectively. The membrane was washed three times with PBST and then incubated with HRP-conjugated secondary antibody for one hour at 37 °C and then washed three times with PBST and once with PBS. Reactive protein bands on the membrane were visualized using ECL reagents (Transgen, Beijing, China) in a dark room. Image grayscale analysis in Image J software was used to compare the density (corresponding to intensity) of the bands on the Western blot, and the resulting data were used to construct a histogram. The intensity of expression of a particular protein band is normalized to the GAPDH band. Other antibodies, such as RAD17, RAD1, CHK1, and HUS1 were purchased from BOSTER (Wuhan, China) according to sequence homology, with all homology exceeding 80%.

### 4.8. Immunofluorescence (IF)

Paraffin sections of different developmental stages tissues (0 h, 15 h, 7 days, and 10 h) were cut at 8 μm thickness. After being dewaxed and hydrated, they were fixed for 10 min in 4% paraformaldehyde, washed by PBS with 0.2% TritonX-100 for 10 min. Rabbit anti–Ac–RAD9 antibody was added and diluted 1:20 with PBST and then incubated overnight at 4 °C. On the next day, sections were incubated in Cy3-conjugated goat anti-rabbit IgG (1:30 dilution; Proteintech) for one hour at 37 °C in the dark, followed by washing with PBST. The samples were then stored in a mounting medium containing DAPI (4′, 6–diamidino–2–phenylindole; ZSGB–BIO, Beijing, China) and examined under a confocal laser microscope.

### 4.9. RNA Interference

According to the cloned *A. sinica* rad9 gene full-length, RNA probes (Sense siRNA, AntiRNAi) were designed online, and synthesized by Takara. The 0 h cyst was deshelled with 50% NaClO. Before the electroporation experiment, the cysts were placed in an electroporation buffer with 400 V shock for 1 s using an EC100 electroporator. The cysts containing 4 mM double-stranded RNA in the electroporation buffer was used as the experimental group, and the control group was not included. Subsequently, the shock *Artemia* cyst was added to seawater, and cultured in a constant temperature incubator every 5 h. The time points were 5 h, 10 h, 15 h, and 20 h, respectively. Animals were collected for qPCR and photographed every 5 h. 

## 5. Conclusions

A 1238 bp full-length cDNA of *As*–RAD9 (GenBank accession number: MH797557) was obtained, which contained an 1131 bp ORF, encoding a 376-amino-acid protein. We determined that the changed mRNA and protein expression level of *As*–RAD9 is related to the cell cycle reactivation during diapause embryo restarting. To further understand the functions of the 9–1–1 complex and the involved signaling pathway, expression levels of *As*–RAD9, *As*–RAD1, *As*–HUS1, *As*–RAD17, and *As*–CHK1 were analyzed by Western blotting. Results showed that the expression levels of these proteins increased significantly at 0–10h, probably due to the rapid embryo reactivation, accelerated mitosis, expanded DNA replication, and RNA transcript, leading to an increase in the expression of cell cycle related proteins. After 15h, the expression levels of these proteins were decreased. This may be due to the mitosis process going stable, and the DNA damage or mispairing may be weakened. The transcriptional and protein expressions of *As*–RAD9 were highly upregulated when the temperature was lowered, and downregulated when the salinity rose up, which indicates that *As*–RAD9 may be involved in the different signaling pathway under different stresses. The RNA interference assay further suggested that *As*–RAD9 is necessary for diapause embryo restarting and early embryo development in *A. sinica*. Our current study has provided a new reference for further study of RAD9 in invertebrates.

## Figures and Tables

**Table 1:** Predicted phosphorylation sites in *As*–RAD9.

Name	Position	Context ^a^	Score ^b^	Name	Position	Context ^a^	Score ^b^
Ser	2	---ASHLPY	0.002	Thr	27	GLLQTATKQ	0.025
	41	PVVESKWRT	0.350		29	LQTATKQAE	0.038
	59	WNFISGIQY	0.004		45	SKWRTLEAF	0.100
	68	LAGLSTLPG	0.007		69	AGLSTLPGN	0.028
	78	PAIASLMAF	0.003		83	LMAFTASIT	0.067
	85	AFTASITSP	0.0079		87	TASITSPLT	0.046
	88	ASITSPLTT	0.662		91	TSPLTTQHT	0.096
	113	LAPPSAASA	0.032		92	SPLTTQHTL	0.192
	116	PSAASAFVG	0.114		95	TTQHTLLFN	0.005
	130	AAVGSIGLG	0.030		165	EMPSTEDLV	0.547
	159	FKVMSGEMP	0.956		221	HVSPTHYVP	0.212
	164	GEMPSTEDL	0.996		234	AARVTQILS	0.174
	177	PAILSPGAL	0.069		241	LSSLTITQL	0.037
	213	IAFASRGNH	0.046		243	SLTITQLLK	0.015
	219	GNHVSPTHY	0.294		259	EDCSTPC--	0.382
	227	YVPESDAAA	0.305	Tyr	6	SHLPYIEQG	0.683
	238	TQILSSLTI	0.003		63	SGIQYLAGL	0.127
	239	QILSSLTIT	0.157		144	ILAGYGAGV	0.008
	258	NEDCSTPC-	0.116		223	SPTHYVPES	0.714

^a^ The sequences surrounding the phosphorylation sites. ^b^ The likelihood of the phosphorylation site being real.

**Table 2:** Oligonucleotide primers used in this study.

Primer	Sequence(5’–3’)
RAD9F	TGGTGATTACATTTACTTTG
RAD9R	CGGCACATCAACTACATCAC
3’RAD9	AGATTTGGCGTTGTGAGGTCTTAC
5’RAD9	GGAAGTAGAGCGGAAAACAGTCAG
RT-rad9F	CTAACCCGAAGTTGGATGCTCT
RT-rad9R	CAGATGGACTTGTTTGCTCGC
*β*-actinF	GTGTGACGATGATGTTGCGG
*β*-actinR	GCTGTCCTTTTGACCCATTCC
ORF-rad9F	CCGGAATTCATGGGGAGCGCAAGAATTTT
ORF-rad9R	ACGCGTCGACTTAATCTTCATCTGAATCAA
SiRNAA1	CCTACGAGCAAGAAACAAT

## Supplementary Materials

The following are available online at https://www.mdpi.com/2073-4425/10/10/768/s1, Figure S1: (A) Nucleotide sequence and deduced amino acid sequence of Rad9 gene in A. sinica. (B) Results of domain analysis of putative As-RAD9 protein. Figure S2: Multiple sequence alignment of As-RAD9 protein. Sequence alignment of known RAD9 sequences from 13 species. The sequences and their accession numbers are as follows: the mouse Mus musculus RAD9 (MmRAD9), NP_035367.1; Cricetulus griseus RAD9 (CgRAD9), XP_003509986.1; human Homo sapiens RAD9 (HsRAD9), NP_004575.1; Pan troglodytes RAD9 (PtRAD9), XP_016776852.1; chicken Gallus gallus RAD9,GgRAD9, NP_998748.1; Aquila chrysaetos canadensis RAD9 (AccRAD9), XP_011599156.1; Xenopus tropicalis RAD9 (XtRAD9), NP_001005810.1; Danio rerio RAD9 (DrRAD9), NP_956501.2; Oryzias latipes RAD9 (OlRAD9), XP_004073282.1; silkworm Bombyx mori RAD9 (BmRAD9), XP_004926904.1; Bombus terrestris RAD9 (BtRAD9), XP_020723628.1; A.sinica RAD9 (AsRAD9), MH_797557; Daphnia magna RAD9 (DmRAD9), KZS14537.1. The sequence of the RAD9 domain is shown in red. Figure S3: Phylogenetic tree constructed by RAD9 proteins. The sequence and registration number of the RAD9 are the same as in the legend of Figure S2. The red diamond indicates As-RAD9 from *A. sinica*.

## Abbreviations

*As*-rad9	rad9 mRNA from *Artemia sinica*
*As*-RAD9	RAD9 Cell cycle checkpoint control protein from *Artemia sinica*
*As*-RAD17	RAD17 checkpoint clamp loader component protein from *Artemia sinica*
*As*-RAD1	RAD1 checkpoint DNA exonuclease protein from *Artemia sinica*
*As*-CHK1	serine/threonine protein kinase CHK1 from *Artemia sinica*
UTR	untranslated region
ATR	ataxia- telangiectasia
TOPBP1	DNA topoisomerase II binding protein 1
ATRIP	ATR interacting protein.
Dpb11	DNA replication regulator DPB11
