# Supplementary material for: Investigation of the Possible Role of RAD9 in Post-Diapaused Embryonic Development of the Brine Shrimp Artemia sinica
Source: Genes (Basel). 2019 Sep 30;10(10):768. doi: 10.3390/genes10100768 (PMC6826366; doi:10.3390/genes10100768)
Supplement: Supplementary file 1 [file genes-10-00768-s001.pdf]

A

```
1    AACATGGGGAGCGCAAGAATTTTAAAAAATCTTCCAAATGAGCTACAAAATCTGTTCAAAATGAAGTGCGCTTTACCGTCGCCAAGTGTT
1       M  G  S  A  R  I  L  K  N  L  P  N  E  L  Q  N  L  F  K  M  K  C  A  L  P  S  P  S  V
91   CGGATATTCGTGAGAGTTATACATGCACTT GCTAAGCTTGGTGATTACATTTACTTTGAAGCTGAGAAAGAACAACTTGTCATCAAGACT
30   R  I  F  V  R  V  I  H  A  L  A  K  L  G  D  Y  I  Y  F  E  A  E  K  E  Q  L  V  I  K  T
181  GTTAATTCATCCAGATCAGTTTTTGTTGTTTTTAAACTTTCCAAACAGTTTTTCATTTCCTACGAGCAAGAAACAATTGCTCTTCCTCAA
60   V  N  S  S  R  S  V  F  V  V  F  K  L  S  K  Q  F  F  I  S  Y  E  Q  E  T  I  A  L  P  Q
271  ATATGTAAACTTACTTCGAAGTCTCTGCTGACTGTTTTCCGCTCTACTTCCTTAATTGACAAGTCTGTTGAAACGTGTTTAATTACAATG
90   I  C  K  L  T  S  K  S  L  L  T  V  F  R  S  T  S  L  I  D  K  S  V  E  T  C  L  I  T  M
361  CCAAATGATATGAGTGAATTAGCTATTCAGTTGAGATGCAGATTTGGCGTTGTGAGGTCTTACAACTTGAATATTGTTGAATGTGATGTA
120  P  N  D  M  S  E  L  A  I  Q  L  R  C  R  F  G  V  V  R  S  Y  N  L  N  I  V  E  C  D  V
471  GTTGATGTGCCGTATTCAAGAAATATACTGACTATTGATGAAGTGACTTTGGTGCTAGAGCCAACTAAAATAGCAATGAAGAACTACGTT
150  V  D  V  P  Y  S  R  N  I  L  P  F  H  I  G  A  S  S  K  T  F  S  E  A  V  L  N  F  R  N
561  GATGATATTCAAGACCCTAGGAAAGCAATTCATACCGAGCTTTCGCTCTCAAAAGACGAATTTATTGATTACACATGCAAACAAGGTGTG
180  T  I  D  E  V  T  L  V  L  E  P  T  K  I  A  M  K  N  Y  V  D  D  I  Q  D  P  R  K  A  I
651  GACTTAACTTTCAATCTAAAAGATTTCCGAGTGTTACTACAGTTCACTGAATTAGCCAATTATTCTATCGACTTAAGATTTGAAACGTCA
210  H  T  E  L  S  L  S  K  D  E  F  I  D  Y  T  C  K  Q  G  V  D  L  T  F  N  L  K  D  F  R
741  GGCGATCCGATTGTAGCAAGTGTGGACTATGAACCTTTATTCACTGCGGACTTCGTTTTGGCGACGCTGGATGTAACAAATGTTACTCAG
240  V  L  L  Q  F  T  E  L  A  N  Y  S  I  D  L  R  F  E  T  S  G  D  P  I  V  A  S  V  D  Y
831  CCAACGCCACCCCCTATTCGTGACGCTTTAAATGCTTCTAATATCAATATTAGCTCTGCGAAAACCCCTCGTTCCCAAAGGAAAAGATAC
270  E  P  L  F  T  A  D  F  V  L  A  T  L  D  V  T  N  V  T  Q  P  T  P  P  P  I  R  D  A  L
921  CCGACATCTGTTACTAATTCTGAAGGTGCCCCCATGGAAAATGGACATGAAACCCCAAGCCAGTCAGTCATTGTTAAGAAAATGAAACGG
300  N  A  S  N  I  N  I  S  S  A  K  T  P  R  S  Q  R  K  R  Y  P  T  S  V  T  N  S  E  G  A
1011 ATCTTCAAGCGTTGTTTTGAGACCTCTTTTTCGCAGCGGATTCAAAGTAATGAACAGATACTTGCACTTGATTCAGATGAAGATTAATTT
330  P  M  E  N  G  H  E  T  P  S  Q  S  V  I  V  K  K  M  K  R  I  F  K  R  C  F  E  T  S  F
1101 GTTTTTCTTTTACGTTTTGTAAACCATGTATGTTTATATTAATATCAGCTGTGATCTGAT TTGTCACTAAAAAAAAAAAAAAAAAAAAAA
360  S  Q  R  I  Q  S  N  E  Q  I  L  A  L  D  S  D  E  *
1191 AAAAAAAA
```

B

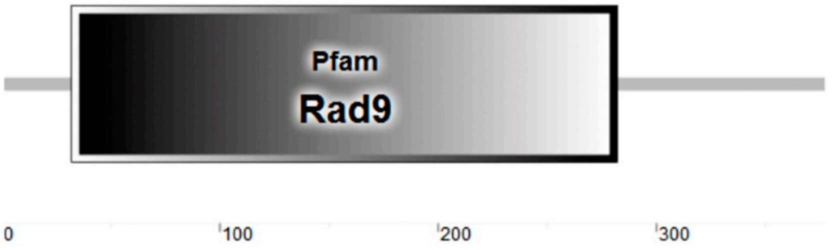

Figure S1. (**A**) Nucleotide sequence and deduced amino acid sequence of Rad9 gene in *A. sinica*. (**B**) Results of domain analysis of putative *As*-RAD9 protein.

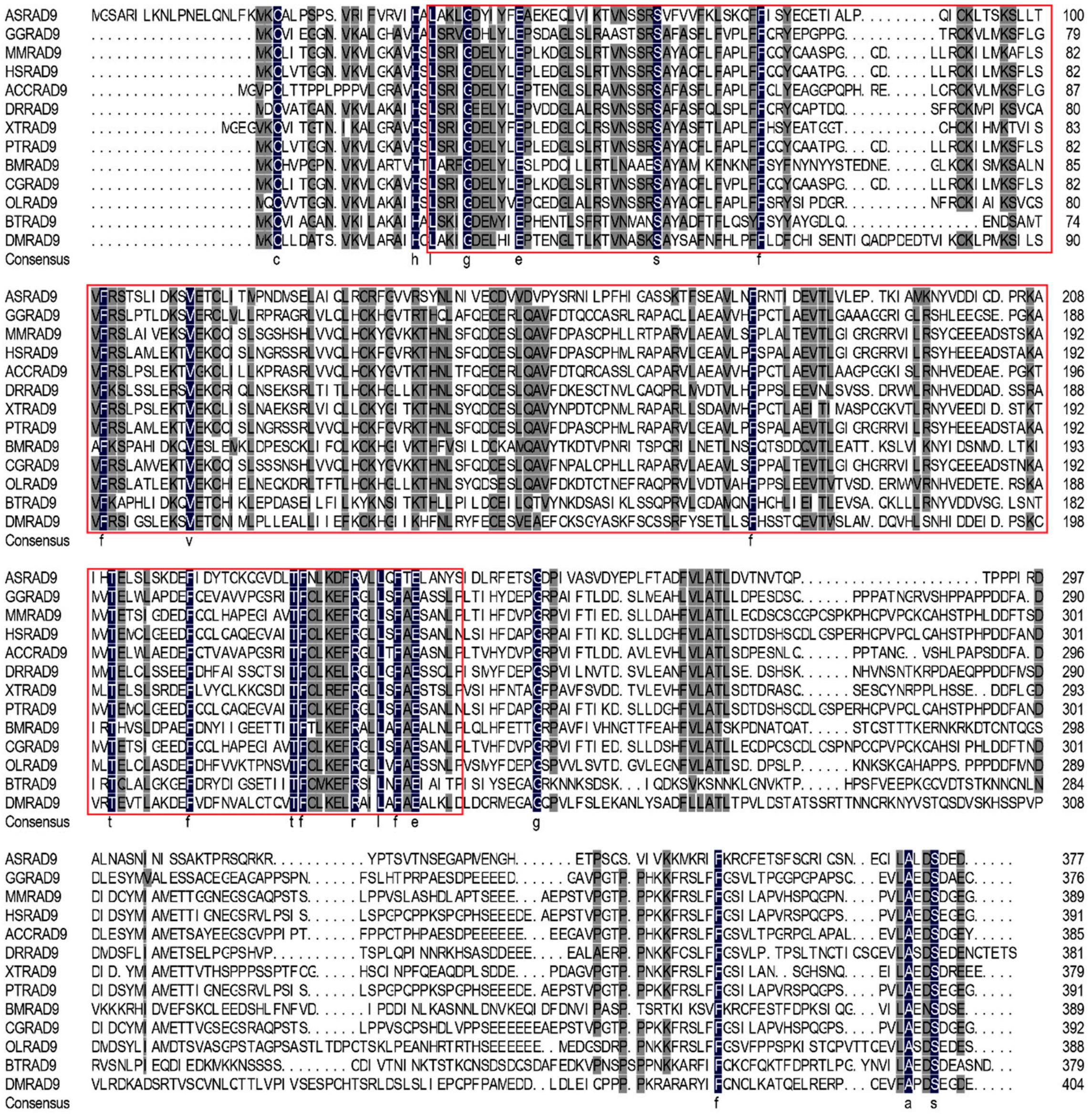

Figure S2: Multiple sequence alignment of *As*-RAD9 protein. Sequence alignment of known RAD9 sequences from 13 species. The sequences and their accession numbers are as follows: MmRAD9, *Mus musculus*, NP_035367.1; CgRAD9, *Cricetulus griseus*, XP_003509986.1; HsRAD9, *Homo sapiens*, NP_004575.1; PtRAD9, *Pan troglodytes*,XP_016776852.1; GgRAD9,*Gallus gallus*, NP_998748.1; AccRAD9, *Aquila chrysaetos canadensis*, XP_011599156.1; XtRAD9,*Xenopus tropicalis*, NP_001005810.1; DrRAD9, *Danio rerio*, NP_956501.2; OlRAD9, *Oryzias latipes*, XP_004073282.1; BmRAD9, *Bombyx mori*, XP_004926904.1; BtRAD9, *Bombus terrestris*,XP_020723628.1; AsRAD9, *Artemia sinica*, (MH_797557); DmRAD9, *Daphnia magna*, KZS14537.1. The sequence of the RAD9 domain is shown in red.

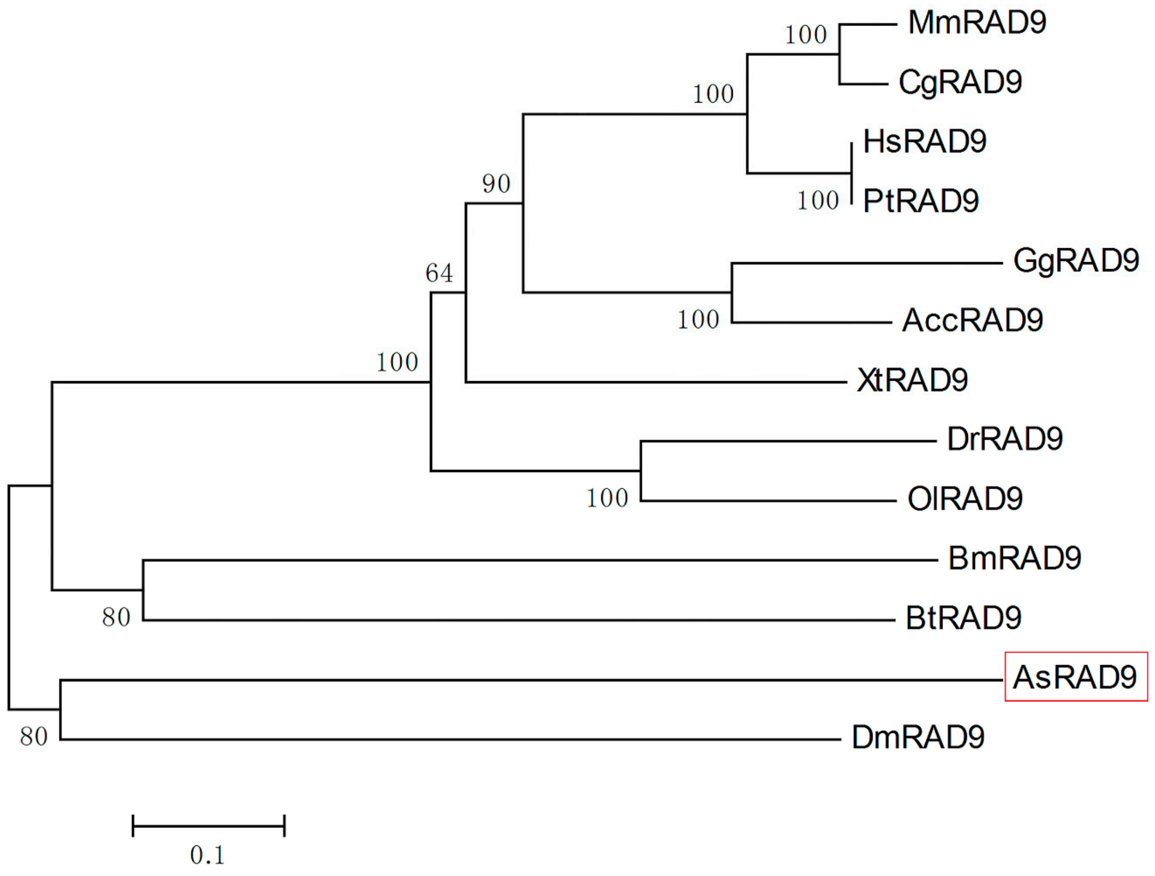

Figure S3: Phylogenetic tree constructed by RAD9 proteins**.** The sequence and registration number of the RAD9 are the same as in the legend of Figure S2. The red diamond indicates *As*-RAD9 from *A. sinica*.
